# An *Escherichia coli* Phosphotransferase System Modulates Methylglyoxal Resistance by Regulating Intracellular Potassium

**DOI:** 10.1111/mmi.70068

**Published:** 2026-04-10

**Authors:** Sara Alexander, Mark Goulian

**Affiliations:** ^1^ Cell and Molecular Biology Graduate Group, Perelman School of Medicine University of Pennsylvania Philadelphia Pennsylvania USA; ^2^ Department of Biology University of Pennsylvania Philadelphia Pennsylvania USA; ^3^ Department of Physics & Astronomy University of Pennsylvania Philadelphia Pennsylvania USA

**Keywords:** *Escherichia coli*, methylglyoxal, pH, phosphotransferase systems, potassium, PTS^Ntr^, pyruvaldehyde

## Abstract

Methylglyoxal is a toxic aldehyde produced during cellular metabolism across all domains of life. To cope with methylglyoxal stress, *Escherichia coli* employs the glyoxalase detoxification pathway coupled with Kef‐mediated potassium/proton antiport. However, the Kef system is protective only when extracellular potassium is well below concentrations typically found in mammalian hosts. The regulatory phosphotransferase system (PTS) that is historically known as the nitrogen‐related PTS (PTS^Ntr^) has previously been shown to modulate potassium homeostasis and other processes in *E. coli* . Here, we identified this regulatory PTS as a mediator of methylglyoxal resistance for potassium concentrations that are comparable to those encountered in the context of host colonization or infection. We found that loss of unphosphorylated PtsN increases survival in methylglyoxal, and that this depended on the potassium/proton antiporter YcgO, whose activity decreases intracellular potassium and pH. While cytoplasmic acidification has been hypothesized to underlie protection from methylglyoxal via potassium/proton antiport, our results suggest the effects of acidification and intracellular potassium cannot be easily separated. Loss of potassium import through Trk increased survival in methylglyoxal and decreased intracellular potassium with only a relatively small decrease in pH. Moreover, the addition of acetate, which acidifies the cytoplasm and protects cells from methylglyoxal, also decreased intracellular potassium. Our results demonstrate that for extracellular potassium levels relevant for host infection and colonization, PtsN modulates methylglyoxal resistance by regulating potassium transport, and that low intracellular potassium, in addition to acidification, could play a direct role in protecting against methylglyoxal stress.

## Graphical abstract

*Escherichia coli* can survive methylglyoxal stress by modulating phosphorylation of a regulatory phosphotransferase system, which, in turn, regulates the activity of a potassium/proton antiporter. The constitutive potassium importer Trk also contributes to intracellular potassium levels. Cells that achieve a low intracellular potassium concentration, which is coupled to intracellular pH, are protected from methylglyoxal‐induced killing. Created in BioRender; https://BioRender.com/ys2f7q0.

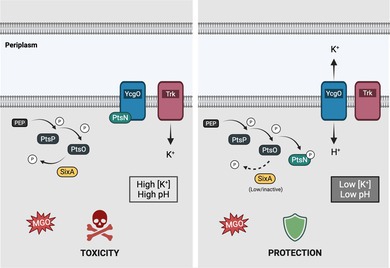

## 1 Introduction

A fundamental challenge for all cells is coping with the inevitable formation of toxic compounds during metabolism (Danchin 2017). Methylglyoxal (MGO) is a highly reactive dicarbonyl that is generated nonenzymatically from various metabolic processes across all domains of life (Thornalley 1996; Chakraborty et al. 2014). MGO has been implicated in infection and host immunity as it is produced by macrophages in response to intracellular bacteria and may play a role in shaping microbial communities in the gut (Anaya‐Sanchez et al. 2025; Baskaran et al. 1989; Akinrimisi et al. 2025). A variety of bacteria, including *Enterobacteriaceae*, also produce MGO enzymatically via methylglyoxal synthase, which is thought to protect against the toxic effects of sugar phosphate accumulation (Hopper and Cooper 1971; Ferguson et al. 1998; Weber et al. 2005; Totemeyer et al. 1998; Kadner et al. 1992). However, MGO itself is toxic to cells due to its propensity to chemically modify DNA, proteins, and other molecules, which can lead to cellular dysfunction and death (Rabie et al. 2016; Yim et al. 1995; Fraval and McBrien 1980; Kang 2003; Thornalley 2008; Cooper and Anderson 1970).

Cells protect themselves from MGO primarily through the glyoxalase system, which facilitates glutathione‐dependent detoxification (Chakraborty et al. 2014; Ferguson et al. 1998). In this pathway, MGO spontaneously reacts with glutathione to form a hemithioacetal that is converted to S‐lactoylglutathione (SLG) by glyoxalase I (GloA or Glo1). Glyoxalase II (GloB or Glo2) then catalyzes the formation of D‐lactate from SLG, which regenerates glutathione (Cooper and Anderson 1970; Cliffe and Waley 1961; Still 1941; MacLean et al. 1998). In *Escherichia coli* and other Gram‐negative bacteria, the detoxification intermediate SLG also activates the potassium (K^+^)‐proton (H^+^) antiporters KefB and KefC, leading to potassium export and proton import, which provides protection from MGO (Ferguson et al. 1998, 1993; MacLean et al. 1998; Ozyamak et al. 2010; Booth 2005). When MGO or other activating electrophiles are absent, Kef transport activity is inhibited by glutathione (Meury and Kepes 1982). A similar MGO protective mechanism by a K^+^/H^+^ antiporter has been demonstrated in *Bacillus subtilis*, where bacillithiol plays a similar role to that of glutathione (Chandrangsu et al. 2014). The resulting cytoplasmic acidification resulting from Kef‐mediated ion exchange reportedly protects from MGO (Ferguson et al. 1995), however, to our knowledge, the mechanism for this protection is not known (Kalapos 1999). In addition, protection from Kef‐mediated potassium transport occurs only when extracellular potassium is very low (≲ 0.2 mM) (Ferguson et al. 1995). It remains unclear whether potassium transport contributes to MGO resistance at higher potassium concentrations, such as those found in animal hosts (Spencer 1959).

**FIGURE 1:**
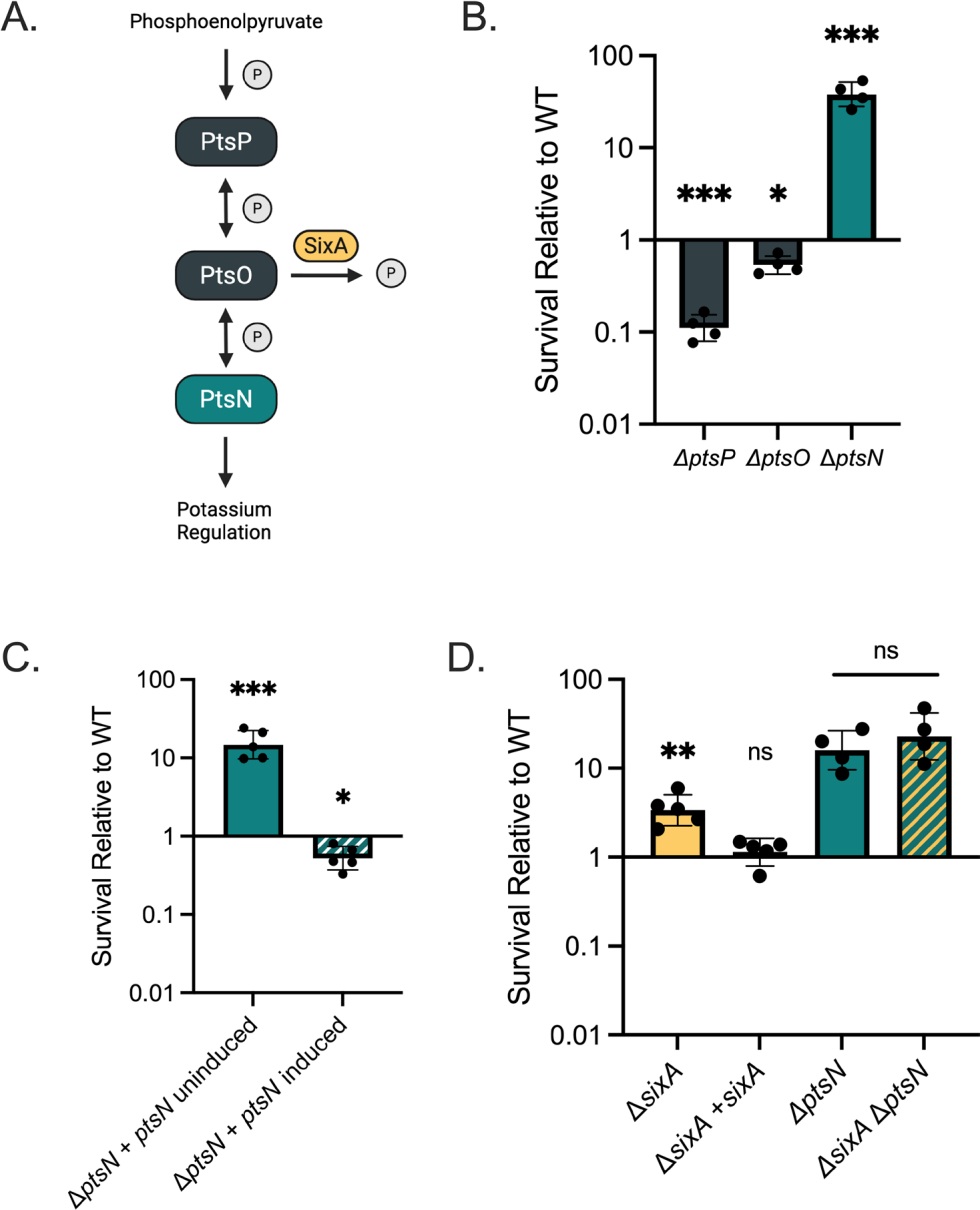
Effect of PTS^Reg^ on sensitivity to MGO. (A) In the PTS^Reg^ pathway, a phosphoryl group is transferred successively from phosphoenolpyruvate to PtsP, then to PtsO, and finally to PtsN. SixA dephosphorylates PtsO. Created in BioRender. (2026) https://BioRender.com/8mggriy. (B) Survival of PTS^Reg^ mutants relative to WT following treatment with 0.8 mM MGO for 1 h in K5 medium. Survival relative to WT is the ratio of the CFU/mL of the indicated mutant after MGO treatment to the CFU/mL at the time of MGO addition, divided by the corresponding ratio for WT (see Section 4). Filled bars represent geometric means; error bars are geometric standard deviations. Significance was calculated using a one‐sample ratio *t*‐test versus a hypothetical value of 1 (*p < 0.05, ***p < 0.001, *n* = 4). (C) Complementation of Δ*ptsN* with a chromosomal *ptsN* transcribed from a tetracycline‐inducible promoter. Expression was induced with 0.1 ng/mL anhydrotetracycline (ATC). The uninduced sample is compared to an uninduced WT control (strain SAA49, Table 1); the induced sample is relative to this WT + 0.1 ng/mL ATC. *p < 0.05, ***p < 0.001, *n* = 5. (D) Effect of *sixA* deletion on MGO sensitivity relative to WT. Significance of Δ*sixA* and the complemented strain, relative to WT, were determined with a one‐sample ratio *t*‐test using a hypothetical value of 1 (**p < 0.01, *n* = 5); the relative survival of *ptsN* and *ptsN sixA* deletion strains were compared with a Welch's unpaired *t*‐test (*n* = 4).

One important modulator of potassium homeostasis in *E. coli* is a regulatory phosphotransferase system (PTS) that is highly conserved across Gram‐negative bacteria and is historically known as the nitrogen‐related phosphotransferase system (PTS^Ntr^) (Barabote and Saier Jr. 2005; Rabus et al. 1999). Despite its name, a direct connection with nitrogen metabolism has never been established. In addition, unlike the classical carbohydrate PTS, this system appears to have a purely regulatory role (Pfluger‐Grau and Gorke 2010). Therefore, in what follows, we will avoid the term nitrogen‐related PTS and instead use the name “regulatory PTS” (PTS^Reg^). In the PTS^Reg^, a phosphoryl group derived from phosphoenolpyruvate is passed from PtsP to the final acceptor PtsN via PtsO (Figure 1A) (Rabus et al. 1999; Pfluger‐Grau and Gorke 2010; Powell et al. 1995; Luttmann et al. 2015). The signals that modulate the phosphorylation levels of these proteins are not well understood. Carbon and nitrogen availability have been suggested to play a role (Lee et al. 2013), but this has not been supported by other studies (Luttmann et al. 2015; Jahn et al. 2013). In addition, the phosphatase SixA dephosphorylates PtsO. Therefore, factors that affect SixA expression or activity could also contribute to PTS^Reg^ phosphorylation and subsequent PtsN regulatory activities (Schulte and Goulian 2018; Schulte et al. 2021).

PtsN interacts with and modulates various proteins and processes based on its phosphorylation state, such as sigma factor selectivity, motility, phosphate‐related stress, the stringent response, and potassium homeostasis (Pfluger‐Grau and Gorke 2010; Bowlin et al. 2022; Luttmann et al. 2012; Lee et al. 2010; Gravina et al. 2021; Karstens et al. 2014; Ronneau et al. 2016). Among these, the regulation of potassium transport by PtsN is the most well‐established in *E. coli* . Unphosphorylated PtsN stimulates expression of the KdpFABC high‐affinity potassium uptake system. Kdp expression is activated when extracellular potassium is very low, but might also be affected by other cations or through cross‐regulation (Luttmann et al. 2009; Epstein 2016; Schramke et al. 2017). Conversely, unphosphorylated PtsN has also been shown to regulate potassium export by directly interacting with and inhibiting the potassium‐proton antiporter YcgO (also known as CvrA), thereby suppressing potassium efflux (Sharma et al. 2016; Patidar et al. 2025). Additionally, there is biochemical evidence that PtsN interacts with the TrkA subunit of the constitutive Trk potassium import system (Lee et al. 2007). However, evidence for a physiological role of this interaction is lacking (Sharma et al. 2016).

Here, we investigate the role of potassium transport in MGO sensitivity for extracellular potassium concentrations that are comparable to those found in the mammalian host environment (5 mM), and thus distinct from the potassium‐limiting conditions that were characterized in previous *E. coli* studies. We identify PTS^Reg^ as a novel regulator of MGO resistance. Specifically, we find that unphosphorylated PtsN sensitizes cells to MGO by inhibiting the potassium/proton antiport activity of YcgO. Relief of this inhibition results in YcgO‐mediated potassium efflux and concomitant cytoplasmic acidification. In addition, we demonstrate that disrupting potassium import by the Trk system increases MGO resistance, suggesting that limiting intracellular potassium accumulation, and possibly the resulting subtle decrease in cytoplasmic pH, also provides protection against MGO. Together, our results suggest a model in which modulation of intracellular potassium, achieved indirectly through changes in PtsN phosphorylation or directly through reduced potassium uptake by Trk, controls bacterial survival during MGO stress in extracellular potassium concentrations that *E. coli* encounters in the context of infection.

## 2 Results

### 2.1 PtsN Phosphorylation State Affects MGO Sensitivity

To identify genes that might contribute to MGO resistance in higher potassium concentrations, we took advantage of transposon sequencing data for *E. coli* in a variety of growth and stress conditions (Wetmore et al. 2015). Transposon insertions within *ptsP*, *ptsO*, and *ptsN* showed fitness effects for cells growing in lysogeny broth (LB) containing MGO. We confirmed the reported Δ*ptsN* phenotype in the *E. coli* MG1655 wild‐type (WT) strain following an hour of MGO exposure in LB (Figure S1). Next, we measured the survival of WT and strains lacking *ptsP*, *ptsO*, or *ptsN* (Figure 1A) in modified minimal medium containing 5 mM potassium (K5). We chose this potassium concentration because it is within the range found in mammalian serum (Spencer 1959; Cohn et al. 2000). In these conditions, MGO treatment resulted in significant killing of the WT strain after up to 2 h of exposure (Figure S2). The Δ*ptsP* and Δ*ptsO* mutants demonstrated significantly worse survival relative to WT, while the Δ*ptsN* strain showed significantly better survival (Figure 1B). We complemented the Δ*ptsN* phenotype by expressing *ptsN* under a tetracycline‐inducible promoter. Addition of anhydrotetracycline (ATC) to induce *ptsN* expression prior to MGO exposure restored survival to near WT levels (Figure 1C).

We suspected that PtsN phosphorylation state influences MGO sensitivity. This hypothesis is supported by the significantly worse MGO survival of both the Δ*ptsP* strain, which has previously been shown to result in decreased PtsN phosphorylation (Luttmann et al. 2015), and the Δ*ptsO* strain (Figure 1B). To explore this further, we took advantage of the fact that the phosphatase SixA dephosphorylates PtsO (Figure 1A) (Schulte and Goulian 2018; Schulte et al. 2021). Since deleting *sixA* increases PtsN phosphorylation (Schulte and Goulian 2018), we tested the effect of this deletion on MGO sensitivity. Survival was significantly higher in the Δ*sixA* strain relative to WT following MGO treatment, and this phenotype could be complemented with *sixA* integrated at an ectopic site in the chromosome (Figure 1D). We also determined that the increased MGO resistance conferred by Δ*sixA* was not additive with the effect of deleting *ptsN*, consistent with Δ*sixA* affecting MGO resistance by decreasing PtsN phosphorylation (Figure 1D). Taken together, these results suggest that unphosphorylated PtsN makes *E. coli* more sensitive to MGO.

### 2.2 The Effect of PtsN on MGO Survival Is Dependent on the Potassium Transporter YcgO

**FIGURE 2:**
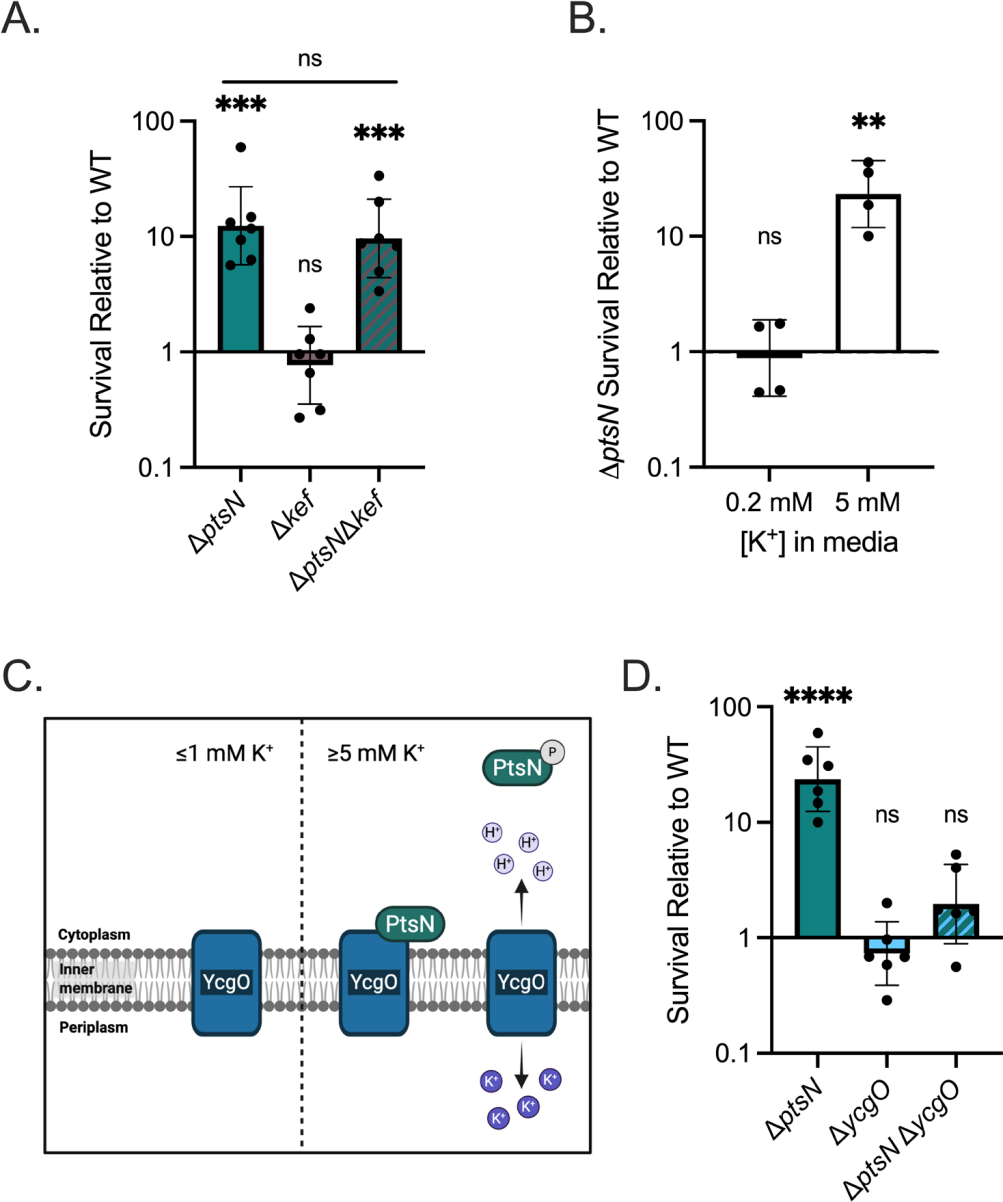
Effect of the Kef and YcgO potassium transporters on PTS^Reg^‐mediated sensitivity to MGO. (A) Survival of strains lacking PtsN and/or both Kef transporters (KefB and KefC, denoted as Δ*kef*) in MGO relative to WT. A one‐sample ratio *t*‐test was used to assess significance of all strains relative to WT using a hypothetical value of 1 (***p < 0.001, *n* = 7); an unpaired *t*‐test was used to compare the relative survival of Δ*ptsN* and Δ*ptsN* Δ*kefBkefC* (*n* = 7). (B) Survival of Δ*kef* relative to WT was compared in media containing 0.2 mM or 5 mM [K^+^] after 1 h of exposure to MGO (one‐sample ratio *t*‐test, hypothetical value = 1, **p < 0.01, *n* = 4). (C) YcgO is inactive when inhibited by binding of unphosphorylated PtsN or when in low external potassium concentrations. Created in BioRender. (2026) https://BioRender.com/ivx5nrp. (D) Survival of *ptsN* and *ycgO* single and double deletions relative to WT in K5 (one‐sample ratio *t*‐test, hypothetical value = 1, ****p < 0.0001, *n* = 6).

As discussed above, potassium transport was reported to increase MGO resistance through the Kef system in potassium‐limiting conditions (Ferguson et al. 1993). However, a role for potassium transport in MGO survival at potassium concentrations relevant for host infection has not been described. Since PtsN has been shown to interact with or regulate the activity of several potassium transporters, we hypothesized that modulation of potassium transport might underlie the enhanced MGO resistance of the Δ*ptsN* strain. We first tested whether Kef plays a role in MGO resistance at 5 mM K^+^. We measured survival after MGO exposure of strains lacking PtsN and/or KefB and KefC. Deletion of both *kefB* and *kefC* did not significantly alter survival relative to WT (Figure 2A). This is consistent with previous results that found KefB/C activity provides protection against MGO in medium containing 0.2 mM K^+^ but not in medium containing 10 mM K^+^ (Ferguson et al. 1995). To rule out the possibility that PtsN inhibits KefB/C in higher potassium conditions, we also tested a mutant lacking both PtsN and the Kef transporters. The Δ*ptsN* Δ*kef* strain demonstrated the same degree of survival in MGO as the Δ*ptsN* strain, confirming that PtsN's effect on MGO sensitivity is not mediated by the Kef system (Figure 2A). Although we ruled out the contribution of Kef, we investigated whether the MGO protection conferred by the loss of PtsN is nonetheless dependent on the concentration of extracellular potassium. To this end, we measured survival of Δ*ptsN* relative to WT in 0.2 mM K^+^, which has frequently been used for MGO survival studies in *E. coli* (Ferguson et al. 1998, 1993, 1995, 1996; Ozyamak et al. 2010). In contrast to the ~10‐fold increase in survival in 5 mM K^+^, the absence of PtsN had no effect on MGO survival in the low potassium medium (Figure 2B). This suggests that the PtsN‐mediated protection is distinct from the canonical Kef pathway and requires sufficient extracellular potassium.

We hypothesized that the recently described potassium transporter YcgO contributes to the enhanced survival of the Δ*ptsN* strain, given that YcgO is a potassium/proton antiporter, it is inhibited by unphosphorylated PtsN, and, consistent with our results in Figure 2B, it is inactive at low extracellular potassium (Sharma et al. 2016; Patidar et al. 2025) (Figure 2C). To test whether the effect of PtsN on MGO sensitivity is through YcgO, we measured survival of a strain lacking YcgO and/or PtsN following MGO exposure. Deletion of *ycgO* alone did not significantly change survival relative to WT, whereas deletion of *ycgO* in the Δ*ptsN* background decreased survival to near‐WT levels (Figure 2D). In addition, deletion of *ycgO* abolished the increased survival of the Δ*sixA* strain (Figure S3). Taken together, these results support a model in which loss of unphosphorylated PtsN increases MGO survival by relieving the inhibition of YcgO.

### 2.3 The YcgO‐Mediated Increase in MGO Resistance Is Associated With Decreased Intracellular Potassium and Cytoplasmic pH

**FIGURE 3:**
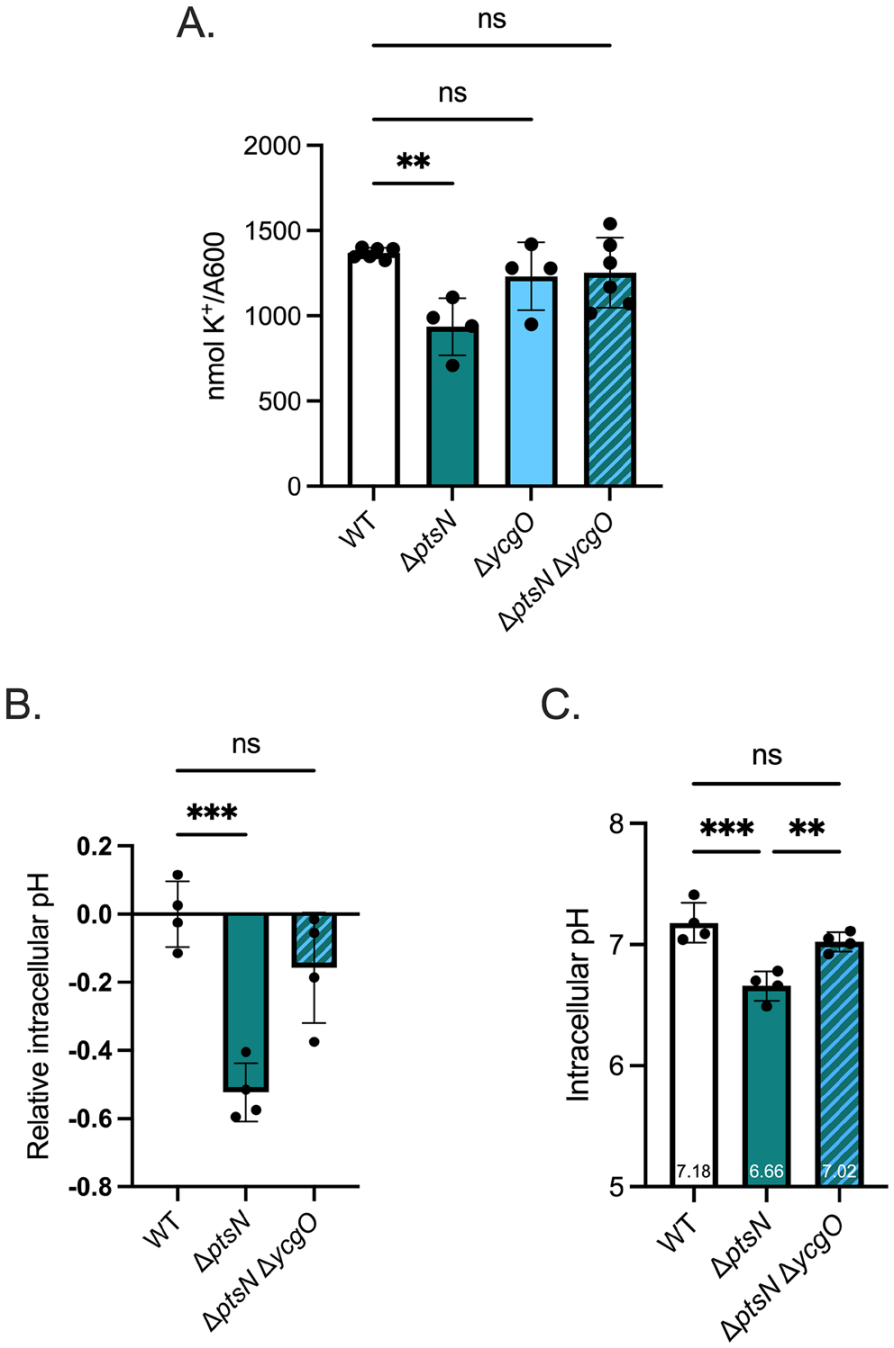
The effect of PtsN and YcgO on intracellular potassium and pH. (A) Intracellular potassium was assayed in exponential‐phase cultures growing in K5 medium by ICP‐OES. Symbols represent the cellular potassium content per optical density at 600 nm (OD_600_) for individual cultures, colored bars represent the averages, and error bars report the standard deviations. Significance was determined by a one‐way ANOVA with Dunnett's test for multiple comparisons (**p < 0.01, *n* = 6 for WT and Δ*ptsN* Δ*ycgO*; *n* = 4 for Δ*ptsN* and Δ*ycgO*). (B) Intracellular pH was measured by treating exponentially growing cells with the pH probe BCECF‐AM and quantifying fluorescence by microscopy (see Section 4). The indicated mutants are derived from the strain NR698, which is used as the WT reference for these experiments (see text and Section 4). Data were normalized to the mean pH of the WT control group for each independent experiment (*n* = 2 per day). Final reported values represent the difference in average pH (ΔpH) of a population of single cells relative to WT, bars represent the mean of each biological replicate, and error bars are standard deviations. One‐way ANOVA was used to assess differences between strains (***p < 0.001, *n* = 4). (C) Absolute intracellular pH values for the experiment described in (B). Intracellular pH was measured as described above; each data point represents the average pH of a population of single cells. Bars represent the mean across biological replicates with the value displayed at the base, and error bars represent standard deviations. Significance was determined by one‐way ANOVA and Tukey's multiple comparisons test (**p < 0.01, ***p < 0.001, *n* = 4).

Previous work found that in a Δ*ptsN* background, YcgO‐mediated potassium transport is active in ≥ 20 mM K^+^ but not in 1 mM K^+^ (Sharma et al. 2016). To determine if PtsN and YcgO affect potassium transport in our growth conditions of 5 mM K^+^, we quantified intracellular potassium in WT as well as Δ*ptsN*, Δ*ycgO*, and Δ*ptsN* Δ*ycgO* strains using inductively coupled plasma optical emission spectrometry (ICP‐OES). The Δ*ptsN* mutant exhibited a significant decrease in intracellular potassium compared to WT, which was restored to WT levels upon deletion of *ycgO* (Figure 3A). The Δ*ycgO* deletion alone, on the other hand, had similar intracellular potassium levels as WT. These results indicate that, in the absence of PtsN, YcgO functions as a potassium transporter in media containing 5 mM K^+^.

Previous studies have suggested that when extracellular potassium is low (0.2 mM), cytoplasmic acidification mediated by Kef potassium/proton antiport provides protection from MGO (Ferguson et al. 1998, 1993, 1995). Therefore, we tested whether YcgO activity in a Δ*ptsN* background affects intracellular pH in 5 mM K^+^. To measure intracellular pH, we used the acetoxymethyl ester of 2′,7′‐bis‐(2‐carboxyethyl)‐5‐(and‐6)‐carboxyfluorescein (BCECF‐AM), which crosses membranes and is converted to BCECF, a membrane impermeable compound that has a pH‐sensitive fluorescence spectrum (Homolya et al. 1993). For these experiments, we used the *E. coli* strain NR698, which has a leaky outer membrane (Ruiz et al. 2005) and was significantly more permeable to BCECF‐AM than was MG1655. We confirmed that PtsN has a similar effect on MGO sensitivity in this strain background (Figure S4). From fluorescence microscopy measurements of cells loaded with BCECF, we found that deleting *ptsN* lowered intracellular pH by approximately 0.5 units relative to WT (Figure 3B,C). On the other hand, the double deletion Δ*ptsN* Δ*ycgO* had an intracellular pH that was not significantly different from that of the parent strain NR698 (Figure 3B,C), consistent with YcgO activity contributing to the decreased pH of the Δ*ptsN* strain.

### 2.4 Parallel Potassium Transport Pathways Confer MGO Resistance Additively

**FIGURE 4:**
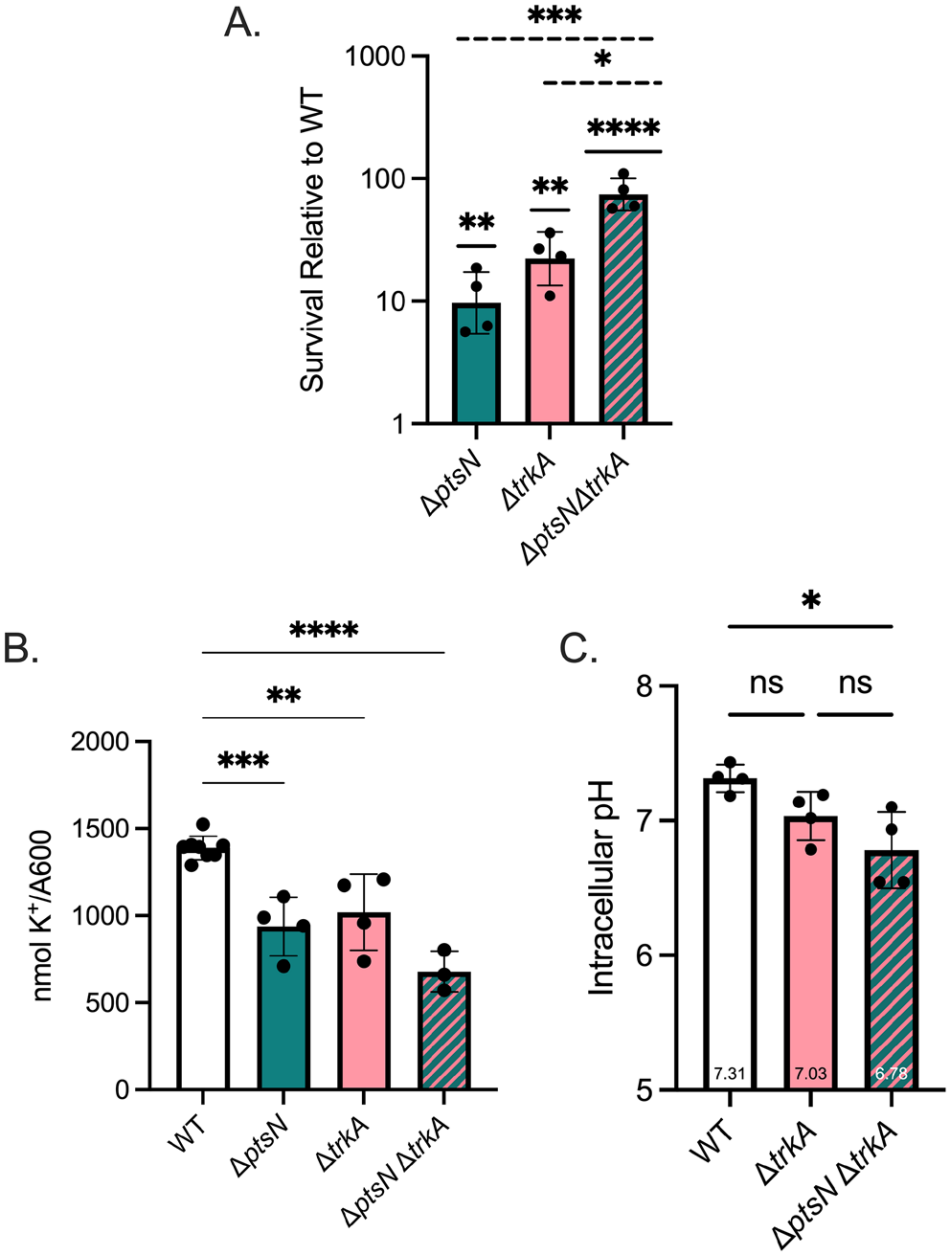
The effect of the Trk potassium import pathway on MGO sensitivity. (A) Survival relative to WT was assessed by a one‐sample ratio *t*‐test with a hypothetical value of 1 (*p < 0.05, **p < 0.01, ****p < 0.0001, *n* = 4). Statistical differences between Δ*ptsN* Δ*trkA* and the respective single mutants were determined by a one‐way ANOVA with log‐normal distribution and Dunnett's multiple comparison test (*p < 0.05, ***p < 0.001, *n* = 4). (B) Intracellular potassium was assessed as in Figure 3. Symbols represent the cellular potassium content per OD_600_ for individual cultures in K5, colored bars represent the averages, and error bars report the standard deviations. One‐way ANOVA was used to assess differences between WT and the deletion strains (**p < 0.01, ***p < 0.001, ****p < 0.0001, *n* = 8 for WT, *n* = 4 for Δ*ptsN* and Δ*trkA*, *n* = 3 for Δ*ptsN* Δ*trkA*). (C) Intracellular pH was measured as in Figure 3. Significance was evaluated by one‐way ANOVA and Tukey's multiple comparisons test (*p < 0.05, *n* = 4).

As noted above, in vitro studies suggested PtsN interacts with the Trk potassium transporter (Lee et al. 2007), but evidence supporting a role in vivo is lacking (Sharma et al. 2016). To investigate whether Trk might contribute to the MGO resistance conferred by Δ*ptsN*, we measured the survival of Δ*ptsN*, Δ*trkA*, and Δ*ptsN* Δ*trkA* relative to WT following treatment with MGO. The Δ*trkA* strain survived significantly better than WT, and this survival was further improved upon deletion of *ptsN* in this background (Figure 4A). This suggests that while Trk affects MGO resistance, it does so independently of PtsN. We also found that the Δ*trkA* mutant had significantly lower intracellular potassium levels relative to WT, and that potassium levels were even lower in the Δ*ptsN* Δ*trkA* strain (Figure 4B). To test whether the Trk potassium transport system affects intracellular pH in *E. coli* , we measured cytoplasmic pH of Δ*ptsN* and/or Δ*trkA* in the NR698 (leaky outer membrane) strain background. We confirmed that loss of TrkA increases MGO resistance in NR698 as in MG1655 (Figure S4). When we measured pH, we found that the Δ*trkA* strain has an intracellular pH approximately 0.3 units lower than WT, although the difference did not meet the threshold for statistical significance (*p* = 0.18) (Figure 4C). As with intracellular potassium, deletion of *ptsN* in the Δ*trkA* background further decreased intracellular pH (Figure 4C). This suggests that the absence of the Trk system protects from MGO toxicity through a decrease in intracellular potassium and possibly pH.

### 2.5 Increased MGO Protection From Sodium Acetate Is Associated With a Decrease in Both Intracellular Potassium and pH

**FIGURE 5:**
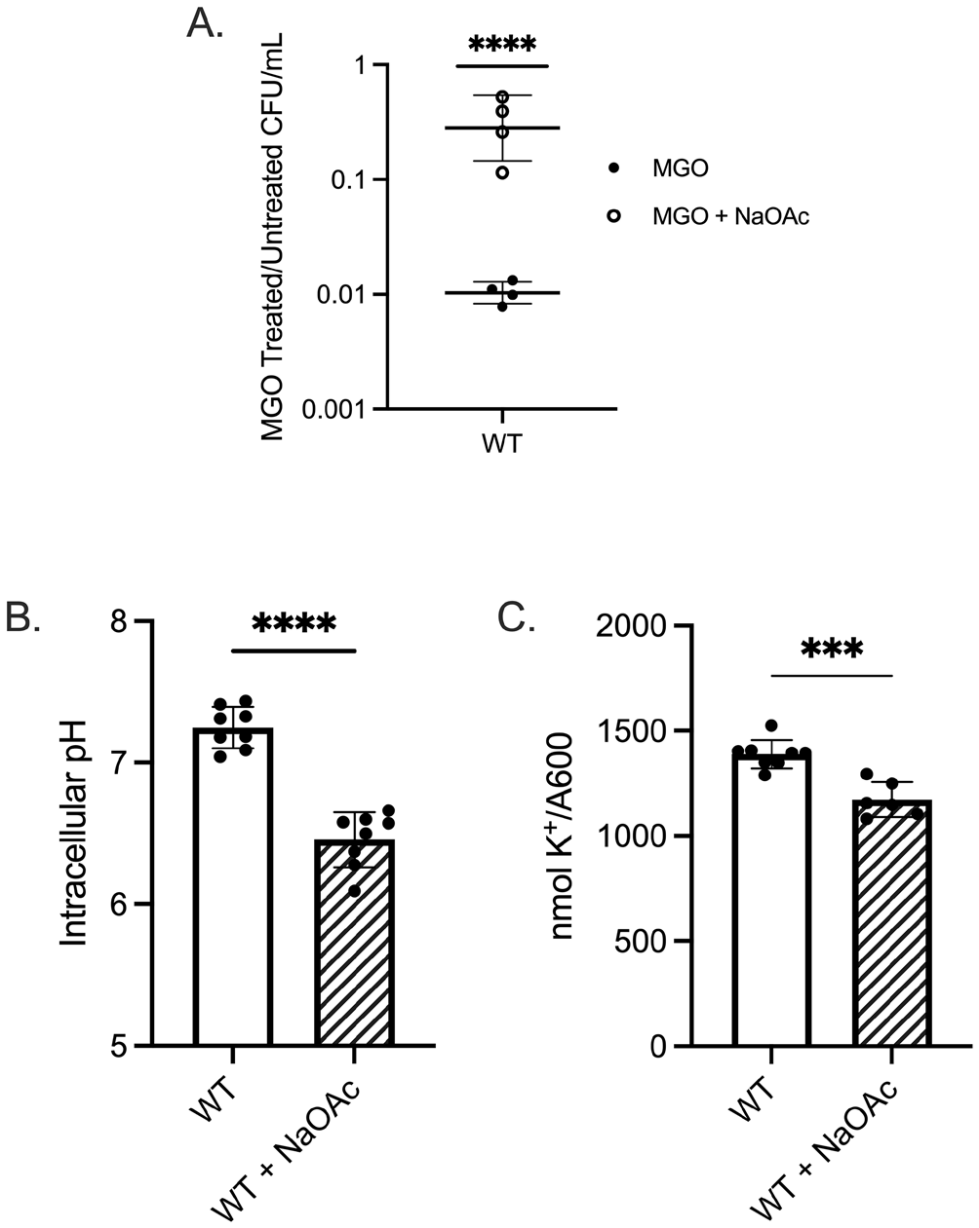
Effect of sodium acetate (NaOAc) on sensitivity to MGO, intracellular pH, and potassium. (A) Where indicated, NaOAc was added to 25 mM at the time of MGO addition. Symbols represent the ratio of treated to untreated CFU/mL for each biological replicate. Closed circles were only treated with MGO, while open circles were also treated with NaOAc. Differences in survival between conditions were assessed using a one‐way ANOVA with the indicated comparisons (****p < 0.0001, *n* = 4). (B) Intracellular pH of strains with or without NaOAc treatment. One‐way ANOVA was used to assess differences between conditions (****p < 0.0001, *n* = 8). (C) Intracellular potassium was measured in WT *E. coli* with or without the addition of 25 mM NaOAc using ICP‐OES. Data presented as nmol K^+^ per OD_600_ at time of culture collection. Welch's *t*‐test was used to assess differences between conditions (***p < 0.001, *n* = 6 for control; *n* = 4 for NaOAc‐treated).

The addition of sodium acetate (NaOAc) to growth medium lowers cytoplasmic pH, since the conjugate acid (acetic acid) crosses lipid bilayers (Warnecke and Gill 2005; Walter and Gutknecht 1984). Even for growth medium buffered to neutral pH, where the fraction of acetic acid is very low, a small decrease in pH can be achieved (Ferguson et al. 1995). This mild acidification protects from MGO‐induced killing and has been used to support the argument for cytoplasmic acidification as the basis of K^+^/H^+^ antiport‐mediated protection (Ferguson et al. 1998, 1995, 1996). To test if acetate is protective in our growth conditions, we measured the effect of treating cells with 25 mM NaOAc during MGO exposure. As previously reported, the addition of NaOAc significantly improved survival of *E. coli* MG1655 upon exposure to lethal MGO concentrations (Figure 5A). To confirm that the addition of NaOAc decreased intracellular pH, we performed pH measurements as described above and found that intracellular pH was lowered from approximately 7.2 to 6.5, a change of approximately 0.7 units (Figure 5B).

A previous study reported that cytoplasmic acidification by addition of NaOAc did not alter intracellular potassium levels of *E. coli*; however, the data were not published (Ferguson et al. 1995). We therefore measured the effect of NaOAc on intracellular potassium in WT cells grown in K5 with and without 25 mM NaOAc. Unexpectedly, we found that NaOAc addition significantly decreased intracellular potassium (Figure 5C). Thus, our results cannot distinguish between a role for decreased intracellular potassium or pH, or perhaps both, in providing protection from MGO.

## 3 Discussion

In this work, we have shown that PtsN serves as a regulator of MGO resistance by modulating potassium efflux (YcgO) in tandem with constitutive potassium import systems (Trk). Previous studies identified a role for potassium transport in modulating MGO resistance through detoxification‐induced Kef activity (Ferguson et al. 1993). However, Kef has been shown to protect *E. coli* from MGO only in very low extracellular potassium concentrations (Ferguson et al. 1993). Indeed, loss of Kef did not significantly change MGO survival in our growth conditions of 5 mM K^+^ (Figure 2A). It is unclear where *E. coli* encounters the potassium limiting conditions in which Kef‐mediated protection would be operative, but it is plausible that it could be important in various ex vivo environments where potassium is scarce, such as in some fresh water sources, soils, or abiotic surfaces (Sardans and Peñuelas 2015; Do and Gries 2021). However, potassium concentrations in animal hosts are significantly higher, with extracellular fluid and the gastrointestinal tract containing an average of 5 mM and 16 mM K^+^, respectively (Spencer 1959). Thus, the MGO resistance pathways uncovered in this study, which function in these host‐relevant potassium ranges, are potentially important in the context of host colonization or infection where cells encounter MGO.

We found that YcgO activity can facilitate protection from MGO in media containing 5 mM K^+^ (Figure 2D); however, the physiological contexts that permit YcgO‐mediated potassium transport remain unknown. Recently, it was demonstrated that unphosphorylated PtsN inhibits YcgO by interacting with the C‐terminal cytoplasmic region of the transporter (Patidar et al. 2025). Under standard laboratory growth conditions, most PtsN protein in the cell is phosphorylated (Luttmann et al. 2015; Schulte and Goulian 2018; Bahr et al. 2011); nevertheless, there is apparently enough unphosphorylated PtsN to effectively inhibit YcgO, as we and others observed no effect of deleting *ycgO* alone on intracellular potassium, or in our case, on MGO resistance (Figures 2D and 3A) (Sharma et al. 2016). However, one would expect that if the ratio of phosphorylated to unphosphorylated PtsN becomes sufficiently high, YcgO inhibition would be relieved. Indeed, eliminating the phosphatase SixA leads to high PtsN phosphorylation and YcgO‐dependent phenotypes, including decreased intracellular potassium (Schulte and Goulian 2018). This aligns with our data showing that loss of SixA confers increased protection from MGO in a YcgO‐dependent manner (Figure 1B and Figure S4).

While deletion of *sixA* serves as a genetic proof‐of‐principal that high PtsN phosphorylation levels can enable YcgO activity, the environmental and cellular signals that modulate PtsN phosphorylation are not well understood. Since SixA dephosphorylates PtsO, conditions that affect SixA's expression or activity have the potential to regulate PtsN phosphorylation. In addition, previous work suggests a role for nitrogen availability through glutamine and alpha‐ketoglutarate‐mediated regulation of PtsP (Lee et al. 2013), although additional studies have not corroborated this observation (Luttmann et al. 2015; Jahn et al. 2013). In some conditions, carbon source availability might also affect PtsN phosphorylation status through changes in phosphoenolpyruvate levels (Brauer et al. 2006), which serves as the phosphoryl group donor to phosphotransferase systems (Figure 1A). Spatiotemporal fluctuations in nutrient availability and other stresses occur throughout the human body; therefore, *E. coli* could encounter conditions that promote MGO resistance through increased PtsN phosphorylation during colonization or infection (Schlomann and Parthasarathy 2019; Chang et al. 2004; Donaldson et al. 2016). More work is needed to identify the factors that govern phosphorylation of PtsN, as well as to determine whether YcgO can function independently of PtsN in certain conditions, in order to further our understanding of YcgO's cellular role and provide deeper insight into how this system modulates protection from MGO.

The constitutive Trk system is one of the major potassium uptake systems in bacteria. In some species, Trk has been implicated in a wide range of processes, including virulence activation (Valente and Xavier 2016), antibiotic resistance (Castaneda‐Garcia et al. 2011; Chen et al. 2004), and stress survival (Vevik et al. 2025; Binepal et al. 2016), but in *E. coli* , little is known about its role in the cell beyond potassium homeostasis (Stautz et al. 2021; Epstein 2003; Bakker and Mangerich 1981; Kuo et al. 2005). Our results identify Trk‐mediated potassium import as a determinant of MGO sensitivity, as deletion of *trkA* significantly improved survival. The loss of Trk was accompanied by a significant decrease in intracellular potassium and a mild decrease in cytoplasmic pH, both of which have been previously reported in *trk*‐deficient *E. coli* (Durand et al. 2016; Hartmann et al. 2022). Since Trk is not a K^+^/H^+^ antiporter, this suggests cytoplasmic potassium can affect pH independently of the transport process. A similar argument applies to the effect of Kdp on pH at very low extracellular potassium (Ferguson et al. 1996). It has been postulated that pH‐mediated protection from MGO relies on cytoplasm pH crossing a particular threshold value (Ferguson et al. 1995, 1996). Thus, it is possible that the subtle decrease in pH observed in the Δ*trkA* strain, while statistically indistinguishable from WT, crossed this threshold for MGO protection. Alternatively, other potassium‐dependent mechanisms besides intracellular pH might also affect MGO susceptibility.

It has long been held that cytoplasmic acidification from potassium/proton antiport activity underlies Kef's protection from MGO (Ferguson et al. 1995). Since YcgO also functions as a potassium/proton antiporter (Patidar et al. 2025), it is likely that the basis for MGO protection is the same for both of these systems. However, to our knowledge, the mechanism underlying how cytoplasmic acidification increases MGO resistance has not been established. Decreased cytoplasmic pH has been suggested to slow or reduce reactions between MGO and macromolecules and/or activate repair systems (Ferguson et al. 1998; Krymkiewicz 1973; Murata‐Kamiya and Kamiya 2001), but whether this occurs in vivo and in response to the relatively small changes in pH associated with Kef and YcgO activity is unclear. The addition of sodium acetate (NaOAc) has been shown to lower intracellular pH and protect cells from MGO (Ferguson et al. 1995), and we observed the same effect (Figure 5A,B). However, we also found that acetate treatment decreased intracellular potassium levels (Figure 5C), which contrasts with previous reports based on unpublished data (Ferguson et al. 1995). Potassium is the major cation in cells and plays numerous biological roles, from maintaining electrochemical potential and turgor pressure to serving as a cofactor for molecular chaperones (Danchin and Nikel 2019). There is also evidence that decreasing intracellular potassium levels can increase protein‐DNA and protein–protein interactions, which could have consequences for repairing damage caused by MGO (Durand et al. 2016; Wood 2011). Thus, it is possible that decreased potassium levels alone, or perhaps in tandem with lower intracellular pH, increase MGO survival. Regardless of the underlying mechanism, our results demonstrate a broader role for potassium transport in protection from MGO, both PtsN‐dependent and independent, than was previously appreciated. Future work will be required to disentangle the effects of intracellular potassium and pH and to determine the underlying protective mechanisms.

## 4 Materials and Methods

### 4.1 Media and Growth Conditions

Strains were grown aerobically at 37°C, except when propagating strains with temperature‐sensitive plasmids, in which case growth was at 30°C. Solid medium consisted of Miller's Lysogeny Broth (LB) agar containing 10% tryptone, 5% yeast extract, 10% NaCl, and 1.5% agar. Minimal media was based on minimal A medium (Miller 1992), but modified to contain different potassium concentrations and buffered with 3‐(N‐Morpholino) Propane‐Sulfonic Acid (MOPS) adjusted to pH 7. For assays with 5 mM K^+^, the growth medium (designated K5) contained, 7.6 mM (NH_4_)_2_SO_4_, 1.7 mM Na citrate, 1 mM MgSO_4_, 2 mM K_2_HPO_4_, 1 mM KH_2_PO_4_, 40 mM MOPS, and 0.2% glucose. For assays with 0.2 mM K^+^, the medium components were identical to those of K5, except the concentrations of K_2_HPO_4_ and KH_2_PO_4_ were 0.08 mM and 0.04 mM, respectively. Methylglyoxal (Alfa Aesar B24664.14) was stored at 4°C with argon in the headspace. When required, ampicillin or kanamycin was added to the growth medium to a concentration of 100 or 50 μg/mL, respectively. To induce expression from the tet promoter for *ptsN* complementation, anhydrotetracycline was added to a final concentration of 0.1 ng/mL from a 1 mg/mL stock prepared in 100% molecular grade ethanol. Phosphate buffered saline (PBS, pH 7.2), which was used to dilute cultures for determining colony forming units (CFU), consisted of 137 mM NaCl, 2.7 mM KCl, 10 mM Na_2_HPO_4_, and 1.8 mM KH_2_PO_4_.

### 4.2 Plasmid and Strain Construction

The strains and plasmids used in this study are listed in Tables 1 and 2, respectively. Phage transduction was performed with P1_vir_ (Miller 1992). FLP recombinase expressed from pCP20 was used to excise FRT‐flanked antibiotic‐resistance cassettes from bacterial genomes (Cherepanov and Wackernagel 1995). Gene deletions from the Keio collection (Baba et al. 2006) were confirmed by PCR. To construct the *ptsN* complementation strain, we constructed a tet‐inducible *ptsN* at the lambda attachment site. First, primers ptsN_tetA_U1 and KEIOP1_GFP_L1 (Table 3), with flanking homology to pAFS07, were used to amplify *ptsN* and the downstream FRT‐KanR‐FRT from SAA60 gDNA (Haldimann and Wanner 2001). Recombineering was then used to insert the sequence into SAA50 harboring pSIM6, replacing *tetA* and *gfp* (Thomason et al. 2023). The resulting construct was confirmed by DNA sequencing and transduced into SAA50 to give SAA68.

**TABLE 1:** Strains used in this study.

Strain	Relevant genotype	References, source, or other information	Associated figures
MG1655 (WT)	F^−^ *ilvG rfb‐50 rph‐1*	*E. coli* Genetic Stock Center (CGSC no. 7740)	1B,D; 2A,B,D; 3A; 4A,B; 5A,C; S1–S3
JES269	MG1655 Δ*ptsN*::(FRT)	P1_vir_(JW3171 × MG1655), treatment with pCP20	1B,D; 2D; 3A; 4A,B; S1
JW3171	Δ*ptsN*::(FRT‐*kan*‐FRT)	Baba et al. (2006)	—
JES215	MG1655 Δ*ptsP*::(FRT)	P1_vir_(JW2408 × MG1655), treatment with pCP20	1B
JW2408	Δ*ptsP*::(FRT‐*kan*‐FRT)	Baba et al. (2006)	—
ARS98	MG1655 Δ*ptsO*::(FRT)	P1_vir_(JW3173 × MG1655), treatment with pCP20	1B
JW3173	Δ*ptsO*::(FRT‐*kan*‐FRT)	Baba et al. (2006)	—
SAA49	MG1655 *att*_λ_::(*cat tetR tetA‐gfp*)	Used as WT control strain; Plasmid pAS07 integrated at *att*_λ_	1C
SAA50	MG1655 Δ*ptsN*::(FRT) *att*_λ_::(*cat tetR tetA‐gfp*)	Used to construct SAA68; Plasmid pAS07 integrated at *att*_λ_	—
SAA60	MG1655 Δ*rapZ*::(FRT‐*kan*‐FRT)	P1_vir_(JW3172 × MG1655), Used to construct SAA68	—
JW3172	Δ*rapZ*::(FRT‐*kan*‐FRT)	Baba et al. (2006)	—
SAA68	MG1655 Δ*ptsN att*λ:(*ptsN*, *kan*, *cat*)	See Section 4	1C
JES13	MG1655 Δ*sixA*::(FRT)	Goulian lab stock, Schulte and Goulian (2018)	1D; S3
JES73	MG1655 Δ(*sixA*) *att*_λ_::(*sixA yfp cat*)	pJS05 integration into JES13 using pINT‐ts	1D
JES194	MG1655 Δ*sixA*::(FRT) Δ*ptsN*::(FRT)	P1_vir_(JW3171 × JES13), treatment with pCP20	1D
JW0046	Δ*kefC*::(FRT‐*kan*‐FRT)	Baba et al. (2006)	—
JW3313	Δ*kefB*::(FRT‐*kan*‐FRT)	Baba et al. (2006)	—
SAA26	MG1655 Δ*kefC*::(FRT)	P1_vir_(JW0046 × MG1655), treatment with pCP20	—
SAA27	MG1655 Δ*ptsN*::(FRT) Δ*kefC*::(FRT)	P1_vir_(JW0046 × JES269), treatment with pCP20	—
SAA29	MG1655 Δ*kefC*::(FRT) Δ*kefB*::(FRT‐*kan*‐FRT)	P1_vir_(JW3313 × SAA26)	2A
SAA30	MG1655 Δ*ptsN*::(FRT) Δ*kefC*::(FRT) Δ*kefB*::(FRT‐*kan*‐FRT)	P1_vir_(JW3313 × SAA27)	2A
JES193	MG1655 Δ*ycgO*::(FRT)	P1_vir_(JW5184 × MG1655), treatment with pCP20	2D; 3A
JW5184	Δ*ycgO*::(FRT‐*kan*‐FRT)	Baba et al. (2006)	—
JES203	MG1655 Δ*ycgO*::(FRT) Δ*ptsN*::(FRT‐*kan*‐FRT)	P1_vir_(JW3171 × JES269)	2D, 3A
JES31	MG1655 Δ*ycgO*::(FRT‐*kan*‐FRT) Δ*sixA*::(FRT)	Goulian lab stock, Schulte and Goulian (2018)	S3
MC4100	F– *araD139* Δ(*argF‐lac*)*U169 rpsL150* (StrR)*relA1 flbB5301 deoC1 ptsF25 rbsR*	Casadaban (1976)	—
NR698	MC4100 *lptD4213*	Ruiz et al. (2005)	3B,C; 4C; 5B; S4
SAA86	NR698 Δ*ptsN*::(FRT‐*kan*‐FRT)	P1_vir_(JW3171 × NR698)	3A; 4C; S4
SAA94	NR698 Δ*ptsN*::(FRT)	SAA86 treated with pCP20	—
SAA98	NR698 Δ*ptsN*::(FRT) Δ*ycgO*::(FRT‐*kan*‐FRT)	P1_vir_(JW5184 × SAA94)	3A; S4
SAA92	NR698 Δ*trkA*::(FRT‐*kan*‐FRT)	P1_vir_(JW3251 × NR698)	4B; S4
SAA99	NR698 Δ*ptsN*::(FRT) Δ*trkA*::(FRT‐*kan*‐FRT)	P1_vir_(JW3251 × SAA94)	4B; S4
JW3251	Δ*trkA*::(FRT‐*kan*‐FRT)	Baba et al. (2006)	—
JES295	MG1655 Δ*trkA*::(FRT‐*kan*‐FRT)	P1_vir_(JW3251 × MG1655)	4A,B
SAA12	MG1655 Δ*ptsN*::(FRT) Δ*trkA*::(FRT‐*kan*‐FRT)	P1_vir_(JW3251 × JES269)	4A,B

**TABLE 2:** Plasmids used in this study.

Plasmid	Relevant genotype	References or construction
pCP20	λ*c*I857(ts) λ*p*_R_‐FLP *repA101*(ts) *oriR101 bla cat*	Cherepanov and Wackernagel (1995)
pINT‐ts	LAM*c*I857(ts), *repA101*(ts), *oriR101*, lpR‐int, *bla*(AmpR)	Haldimann and Wanner (2001)
pSIM6	miniλ recombineering plasmid, pSC101 origin, *repAts*	Datta et al. (2006)
pAS07	pCAH63 *tetR* ^+^ Φ(*tetA* ^+^‐*cfp* ^+^)	Lasaro et al. (2014)
pCAH63	*oriR*_*λ*_ *cat attP*_λ_ *P*_*synl*_ *‐uidAf*	Haldimann and Wanner (2001)
pJS05	pCAH63 *cat* P_*sixA*_‐*sixA* ^+^	Goulian lab stock; P_syn1_‐*uidA* was replaced with *sixA*; *sixA* complementation vector comprising the native promoter (including sequence 694 bp upstream of start codon and 68 bp downstream of stop codon) and a promoterless *yfp*.

**TABLE 3:** Primers used in this study.

Primer	Sequence	Resulting construct and purpose
ptsN_tetA_U1	CATTGATAGAGTTATTTTACCACTCCCTATCAGTGATAGAGAAAAGTGAAatgACAAATAATGATACAAC	*ptsN* Δ*rapZ* FRT‐*kan*‐FRT with flanking homology to pAS07; Recombineering.
KEIOP1_GFP_L1	TGTCAAACATGAGAATTCGTACGGCCGACTAGTAGGTCAGCTAATTAAGCTGTAGGCTGGAGCTGCTTCG	*ptsN* Δ*rapZ* FRT‐*kan*‐FRT with flanking homology to pAS07. Recombineering, sequencing SAA68.

### 4.3 MGO Survival Assays

Overnight cultures grown in K5 were diluted 1:100 into fresh, pre‐warmed K5 and grown to early exponential phase (OD ~0.2). Cells were then diluted 1:10 into pre‐warmed K5 and MGO was added to a final concentration of 0.8 mM and incubated at 37°C. Cultures were exposed to MGO for 1 h, then serially diluted in 1× PBS and 10 μL was plated on LB agar plates and incubated overnight at 37°C. Relative survival was calculated by comparing the ratio of final to initial CFU/mL for each mutant to WT, according to the following formula:

$$\text{Relative survival}=\frac{\;{\left(\frac{\text{final}\;\mathrm{CFU}}{\text{initial}\;\mathrm{CFU}}\right)}_{\mathrm{Mut}}}{{\left(\frac{\text{final}\;\mathrm{CFU}}{\text{initial}\;\mathrm{CFU}}\right)}_{\mathrm{WT}}}$$

For experiments with sodium acetate (NaOAc), a final concentration of 25 mM NaOAc was added immediately prior to the addition of MGO (Ferguson et al. 1995). For *ptsN* complementation experiments, ATC was added at the time of overnight culture dilution to induce *ptsN* expression.

### 4.4 Intracellular Potassium Measurements

Cell pellets for potassium measurements were prepared by spinning through oil as previously described (Schulte and Goulian 2018). Bacteria were grown to early exponential phase (OD ~0.2) in K5, then 1.5 mL of sample was spun through 300 μL of a 2:1 (vol/vol) dibutyl and dioctyl phthalate (Sigma; 524980, 201154) mixture in triplicate for each culture, then spun at 16,000 g. The supernatant was carefully removed, and the remaining pellet was resuspended in 100 μL 1 N nitric acid and digested at room temperature overnight. The next day, 950 μL of diluent (2% nitric acid, 0.5% HCl) was added, samples were centrifuged at 16,000 *g* for 30 min, and 1 mL of supernatant was removed and mixed with 4 mL diluent. Samples were stored at 4°C until analysis. Intracellular potassium measurements were performed on a Spectro Genesis inductively coupled plasma optical emission spectrometer (ICP‐OES). The fully aqueous samples were delivered to the plasma via a modified Lichte maximum dissolved solid nebulizer (MDSN) and cyclonic spray chamber. Instrument drift and performance were monitored using Ar wavelengths 404.442 nm and 430.010 nm. Calibration solutions containing 0.1, 0.5, 1, 5, and 10 mg/L KCl and 0.01, 0.05, 0.1, 0.5, and 1 mg/L NaCl were prepared in distilled H_2_O and analyzed alongside a blank. Potassium was quantified at wavelength 766.491 nm. All data was processed using Smart Analyzer Vision software (v 5.01).

### 4.5 Intracellular pH Measurements by BCECF‐AM

*E. coli* strain NR698 was used for all pH experiments as detailed in the results and Table 1. Intracellular pH was measured using BCECF‐AM as previously described with the following modifications (Aono et al. 1997; Worthan et al. 2024). BCECF‐AM (Invitrogen, B1170) was prepared in anhydrous DMSO (Biotium, 90082) to a final concentration of 1 mM and nigericin (Cayman Chemical, 11437) was prepared in 100% molecular biology grade ethanol to a concentration of 25 mM on the day of pH measurement. NR698 (WT) and derived strains were grown in K5 to exponential phase (OD ~0.2) with aeration at 37°C. For pH calibration, WT (NR698) cultures were clamped in potassium phosphate buffer containing a final concentration of 100 mM potassium and 50 μM nigericin at pH 6, 6.4, 7, 7.4, 8, or 8.4. Next, 10 μM of BCECF‐AM was added and cells were incubated at 37°C in the dark for 60 min. For samples containing NaOAc, a final concentration of 25 mM was added prior to the addition of BCECF‐AM. Following incubation, samples were spun at 6500 g, resuspended in 100 μl K5 or the corresponding pH buffer, and then added to a 1% agarose pad for imaging as previously described (Miyashiro and Goulian 2007). For pH calibration, agarose pads were prepared with the corresponding pH buffer to keep the microenvironment consistent during imaging, otherwise pads were prepared with K5. Fluorescence microscopy was performed with an Olympus IX81 microscope equipped with a 100X UPlanApo NA 1.35 objective lens, a 100‐watt mercury lamp, and a Sensicam QE ccd camera (Cooke Corporation, Romulus, MI). Fluorescence filters, which were used for ratiometric pH measurements of BCECF, were from Chroma (Brattleboro, VT); one filter set (488) consisted of HQ500/20 excitation, Q515lp dichroic, HQ535/30 emission filters, and the other set (440) consisted of D436/20 excitation, 455dclp dichroic, HQ535/30 emission filters. Cells were exposed to filter 440 for 40 ms and filter 488 for 20 ms. Images were analyzed using in‐house software; cells with a mean fluorescence of less than 25 or 50 for 440 and 488 respectively, and with > 8%‐pixel saturation, were removed from analysis. A minimum of 60 cells were analyzed per condition or strain. Ratio of 488/440 calculated for each sample, then average and standard deviation were calculated in Microsoft Excel. Mean fluorescence ratios were fitted to a modified Henderson‐Hasselbalch equation to determine day‐specific calibration parameters using non‐linear least squares regression as described previously (Worthan et al. 2024; James‐Kracke 1992) using python package SciPy (Virtanen et al. 2020).

### 4.6 Statistical Analysis and Figure Preparation

All graphs were prepared and statistical tests performed with GraphPad Prism Version 10.5.0. Details of statistical tests are found in corresponding figure legends. All relevant *p* values were two‐tailed and *p* < 0.05 was considered significant for all analyses and are indicated with asterisks or letters in the text: *p < 0.05, **p < 0.01, ***p < 0.001, and ****p < 0.0001. Figures 1A and 2C were made in BioRender.

## Author Contributions

Sara Alexander: conceptualization (lead); investigation (lead); methodology (lead); writing – original draft (lead); formal analysis (lead); writing – review and editing (lead); visualization (lead). Mark Goulian: conceptualization (supporting); methodology (supporting); supervision (lead); writing – review and editing (supporting).

## Funding

This work was supported by the National Institute of General Medical Sciences (R35GM139541, F31GM154412, and T32 GM07229).

## Disclosure

Copyright Statement: Figures 1A, 2C, and the graphical abstract were created with BioRender.com under publication licenses SG2991T2GQ, PU2991SW2H, and NR2991UDU3. No other third‐party copyrighted materials were used.

## Ethics Statement

The authors have nothing to report.

## Conflicts of Interest

The authors declare no conflicts of interest.

## Supporting information


**Figure S1:** Survival of WT and Δ*ptsN* in MGO during growth in LB. Cultures were exposed to 0.8 mM MGO for 1 h, as described in Section 4. Symbols represent the ratio of treated to untreated (initial) CFUs, bars represent the mean, and error bars represent the standard deviations. Significance was determined by a log‐normal Welch's *t*‐test (***p < 0.001, *n* = 6).
**Figure S2:** Survival of MG1655 in MGO over time. Survival in MGO was assessed in K5 as described in Section 4 for up to 2 h. Symbols represent CFUs after indicated MGO exposure time, bars represent the mean, and error bars are standard deviations. Statistical differences were assessed by log‐normal one‐way ANOVA with Dunnett's multiple comparisons test (**p < 0.01, ***p < 0.001, *n* = 3).
**Figure S3:** MGO resistance of Δ*sixA* and Δ*sixA* Δ*ycgO*. MGO survival was assessed as described in Section 4. Bars represent the geometric mean, and error bars are the geometric standard deviations. Statistical differences were determined by a one‐sample ratio *t*‐test versus a hypothetical value of 1 (*p < 0.5, *n* = 4).
**Figure S4:** Survival of NR698 and derived mutants in MGO. Survival was assessed as described in Section 4. Bars represent the geometric mean and error bars are the geometric standard deviation. Statistical significance was determined by a log‐normal one‐way ANOVA with Dunnett's multiple comparisons test (***p < 0.001, ****p < 0.0001, *n* = 4).

## Data Availability

The data that support the findings of this study will be made available on a public repository.
