# Supplementary material for: An Escherichia coli Phosphotransferase System Modulates Methylglyoxal Resistance by Regulating Intracellular Potassium
Source: Mol Microbiol. 2026 Apr 10;125(6):461–74. doi: 10.1111/mmi.70068 (PMC13445646; doi:10.1111/mmi.70068)
Supplement: Supplementary file 1 [file MMI-125-461-s001.pdf]

SUPPORTING INFORMATION

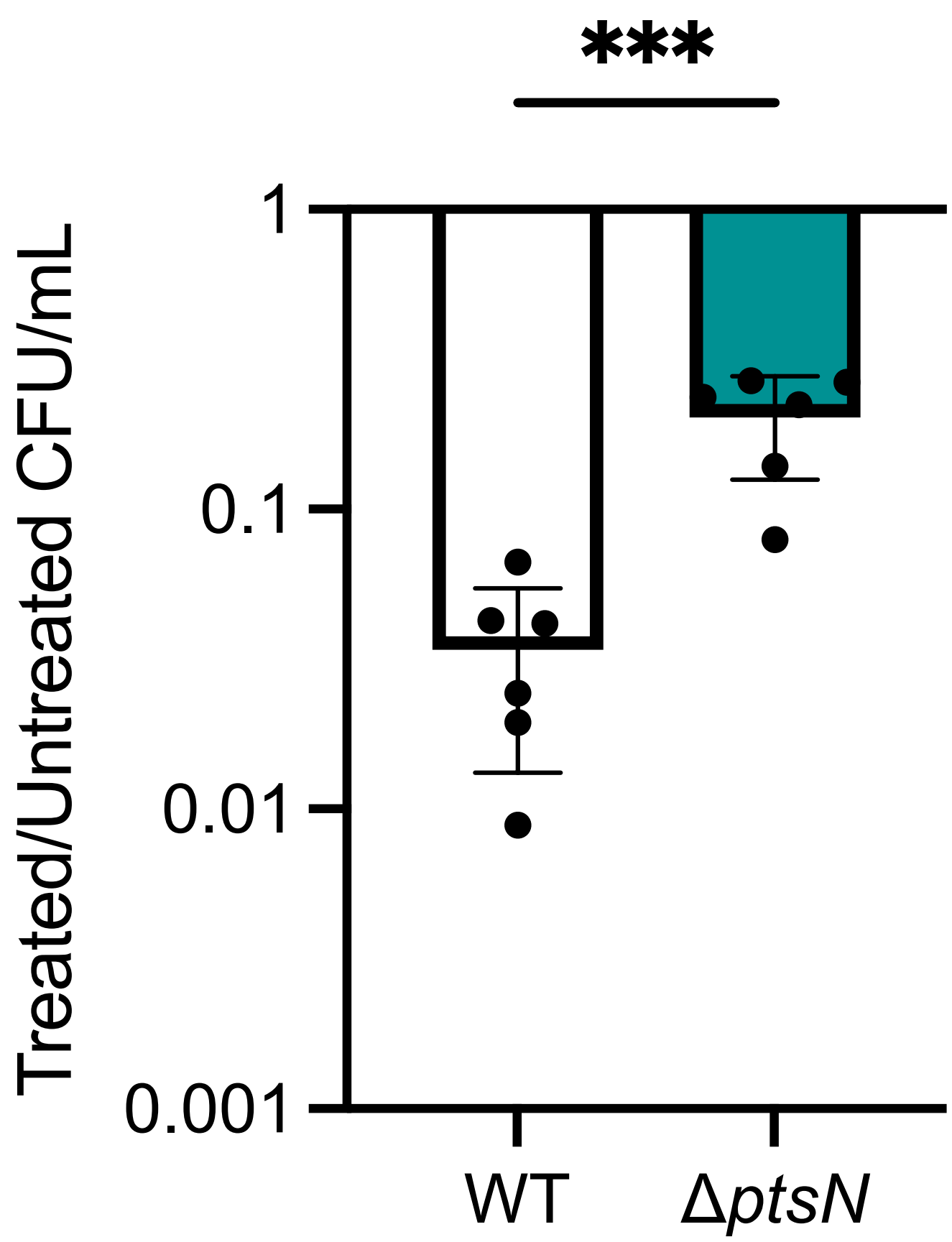

Figure S1. Survival of WT and Δ*ptsN* in MGO during growth in LB. Cultures were exposed to 0.8 mM MGO for one hour, as described in the Materials and Methods. Symbols represent the ratio of treated to untreated (initial) CFUs, bars represent the mean, and error bars represent the standard deviations. Significance was determined by a log-normal Welch's t-test (*** P < 0.001, n = 6).

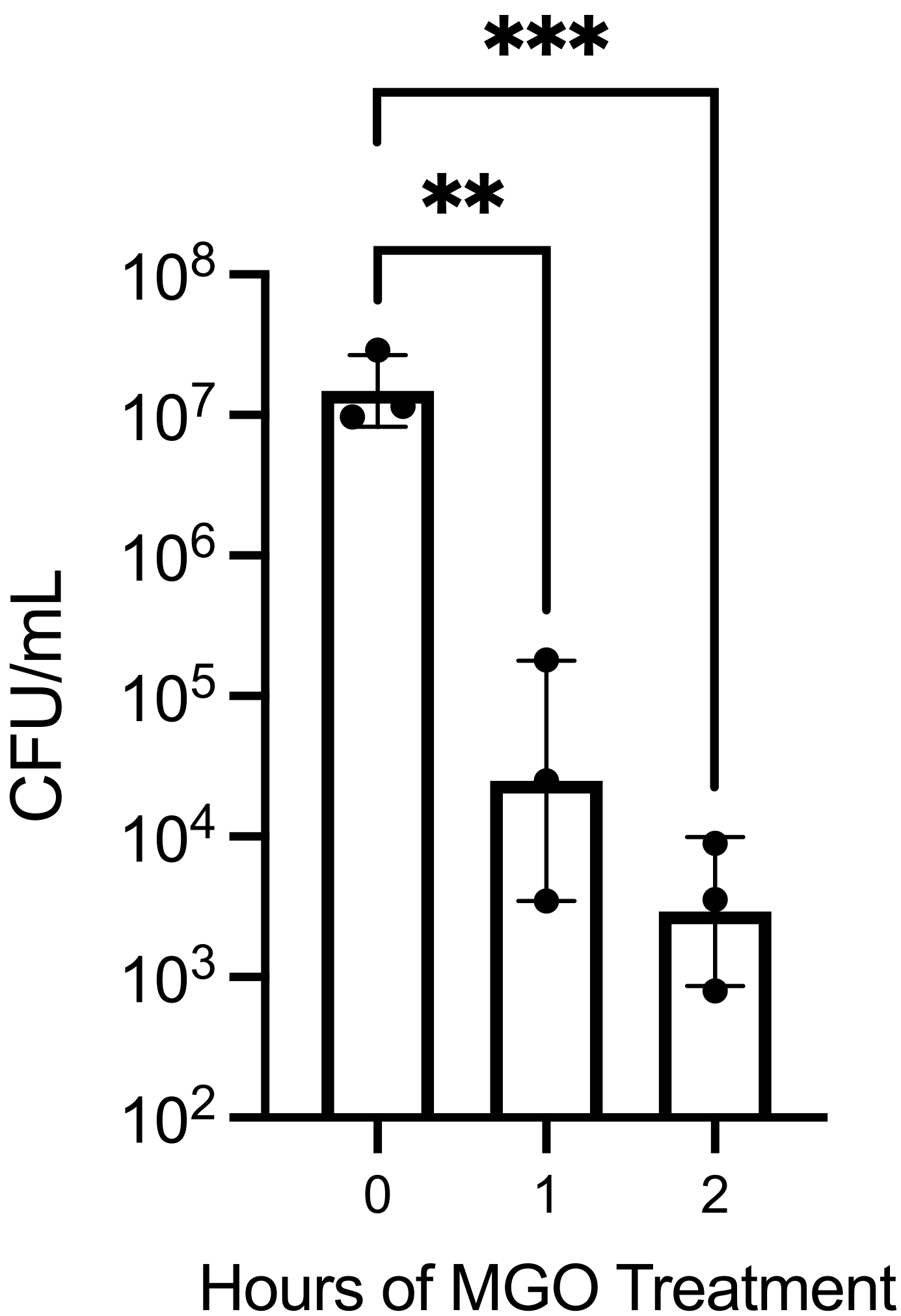

Figure S2. Survival of MG1655 in MGO over time. Survival in MGO was assessed in K5 as described in the Materials and Methods for up to two hours. Symbols represent CFUs after indicated MGO exposure time, bars represent the mean, and error bars are standard deviations. Statistical differences were assessed by log-normal one-way ANOVA with Dunnett's multiple comparisons test. (** $P < 0.01$, *** $P < 0.001$, $n = 3$).

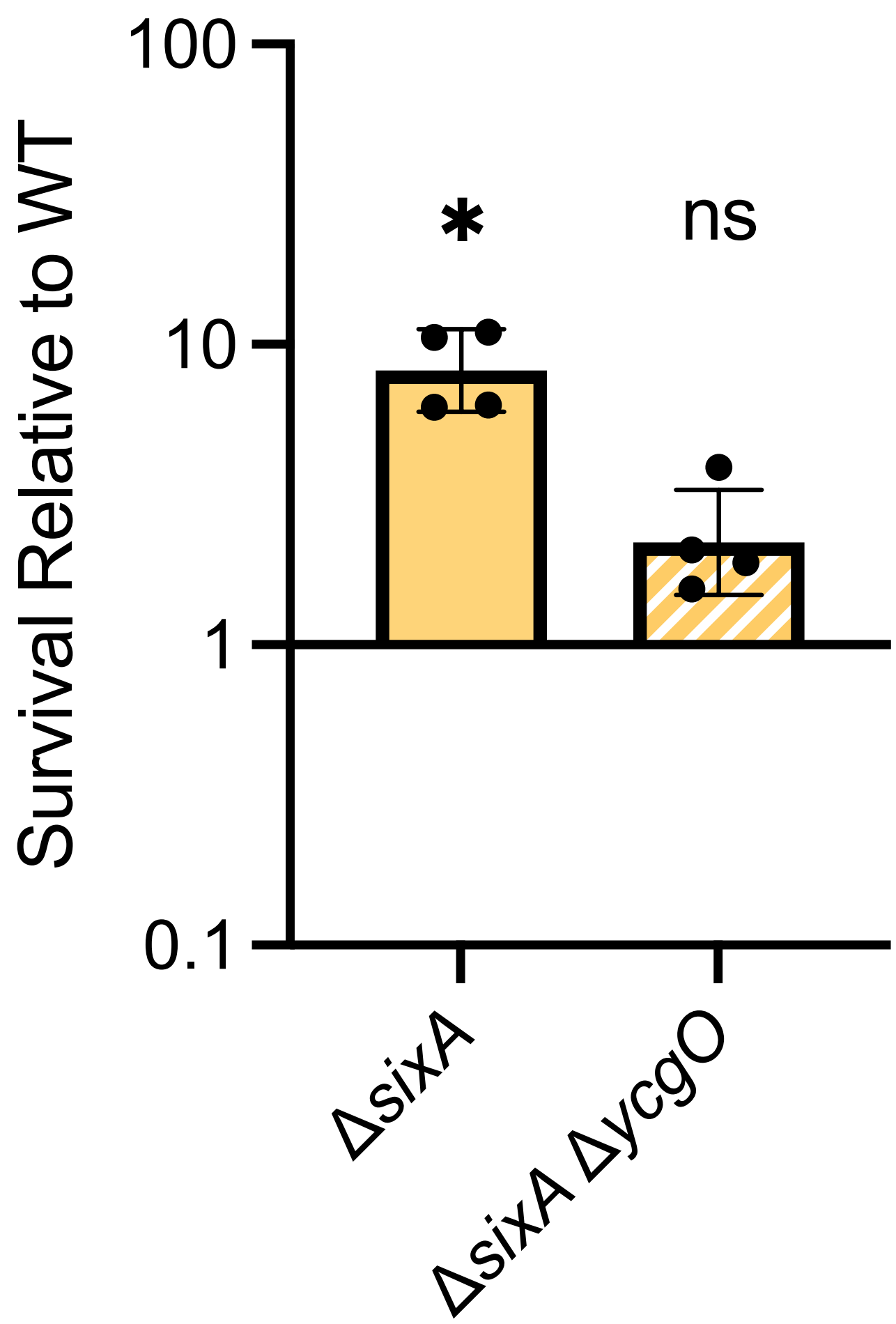

Figure S3. MGO resistance of Δ*sixA* and Δ*sixA* Δ*ycgO*. MGO survival was assessed as described in the Materials and Methods. Bars represent the geometric mean, and error bars are the geometric standard deviations. Statistical differences were determined by a one-sample ratio t-test vs. a hypothetical value of 1. (* P < 0.5, n = 4).

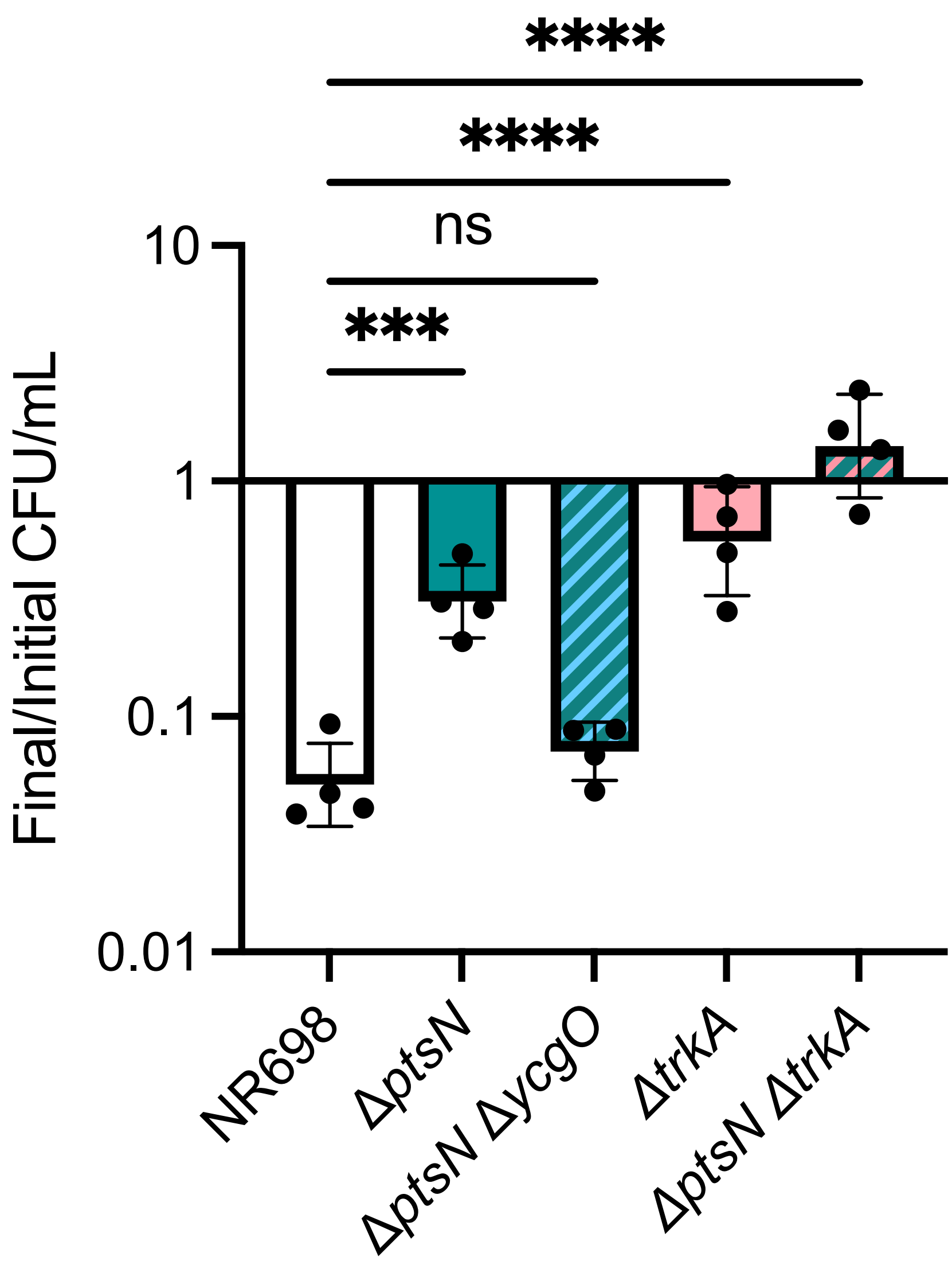

Figure S4. Survival of NR698 and derived mutants in MGO. Survival was assessed as described in the Materials and Methods. Bars represent the geometric mean and error bars are the geometric standard deviation. Statistical significance was determined by a log-normal one-way ANOVA with Dunnett's multiple comparisons test. (*** $P < 0.001$, **** $P < 0.0001$ n = 4).
